# Redefining Criteria to Ensure Adequate Sentinel Lymph Node Biopsy With Dual Tracer for Breast Cancer

**DOI:** 10.3389/fonc.2020.588067

**Published:** 2020-12-03

**Authors:** Li Xu, Jiqiao Yang, Zhenggui Du, Faqing Liang, Yanyan Xie, Quanyi Long, Jie Chen, Helin Zeng, Qing Lv

**Affiliations:** Department of Breast Surgery, West China Hospital, Sichuan University, Chengdu, China

**Keywords:** breast cancer, sentinel lymph node biopsy, radioisotope, methylene blue, 10% rule

## Abstract

**Background:**

For sentinel lymph node biopsy (SLNB) in patients with breast cancer, the dual tracer of blue dye and radioisotope with the 10% rule that all nodes with radioactive count of 10% or more of the hottest node *ex vivo* should be removed is widely accepted. However, the cut-off point of radioactivity is being questioned for possibly excessive removal of negative nodes.

**Methods:**

To compare different percentile rules and optimize the criteria for identifying SLNs, we established a database which prospectively collected the radioactivity, status of blue dye and the pathological results of each SLN in breast cancer patients who successfully underwent SLNB with a combination of methylene blue and radioisotope.

**Results:**

A total of 2,529 SLNs from 1,039 patients were identified from August 2010 to August 2019. 16.4% (414/2,529) positive nodes were removed at a cost of 83.6% (2115/2,529) negative nodes removed excessively. Up to 17.9% (375/2,115) negative nodes were removed as radioactively hot nodes without blue staining. By gradually increasing the threshold by each 10%, the number of negative nodes identified reduced by 18.2% (385/2,115) with only three node-positive patients (1.0%) missed to be identified using the “40% + blue” rule. In patients with ≥ 2 SLNs removed, 12.3% (238/1,942) negative nodes avoided unnecessary removal with only 0.8% (2/239) positive patients missed with the “hottest two + blue” rule.

**Conclusions:**

Our data indicated that the “40% + blue” rule or the “hottest two + blue” rule for SLNB with the dual tracer of blue dye and radioisotope may be considered as a potential alternative rule to minimize extra nodes resected. Nonetheless, it should be validated by prospective trials with long-term follow-up.

## Introduction

The sentinel lymph node (SLN) was discovered in patients with melanoma by Cabanas in 1977 and is defined as the first draining node(s) with a direct lymphatic connection to the primary tumor site (1). Since sentinel lymph node biopsy (SLNB) was first applied to breast cancer by Krag in 1993 to predict the status of axilla and guide further treatment (2), it has become the standard care of the axilla for early stage breast cancer patients with reduced arm morbidities while still offering equivalent survival compared to axillary lymph node dissection (ALND) (3). There are various tracing methods to guide surgeons to identify a sentinel node intraoperatively including blue dye, radioisotope colloid and various novel techniques such as indocyanine green optical imaging and superparamagnetic iron oxide (3). Given the lack of radioisotope and extra requirements for equipment especially in less developed areas, SLNB using single tracer, predominantly blue dye is used in a large number of institutes (4). However, the dual-tracer method combining the radioactive colloid and blue dye with a higher SLN detection rate (>90%) and a lower false negative rate (FNR) (<5%–10%) than either single tracer is constantly recommended in many guidelines such as the 2005 American Society of Clinical Oncology (ASCO) Guideline Recommendations for Sentinel Lymph Node Biopsy in Early-stage Breast Cancer and the 2011 Chinese Anti-Cancer Association (CACA) Guidelines, and is increasingly being applied in many countries and areas such as the United States, Europe, Australia and China (5–7). Most frequently, breast surgeons who use dual tracer of radioisotope and blue dye follow the “10% + blue” rule which was originally proposed by Martin and McMasters that all nodes with a radioactivity count of at least 10% of the hottest node *ex vivo* or blue dye staining should be removed (8).

An ideal criterion of SLN selection should minimize the number of nodes removed, without significantly sacrificing the sensitivity of the procedure. While this approach can reduce the risk of missing positive nodes with a low radioactivity count, it may result in an excessive number of nodes being removed than those identified on lymphoscintigraphy. To seek an ideal cut-off point of a hot SLN, several studies have assessed the validity of the “10% + blue” rule by comparing with other alternative node harvesting rules, including the “50% + blue” rule, the “hottest + blue” rule, and the “4 nodes” rule (9–11). In our institution, we were concerned that excessive number of negative nodes were excised by the “10% + blue” rule. The more SLNs removed, the higher the cost of the procedure for added operative time, pathological charges, medical resources, and most importantly, the long-term complications after surgeries. However, there is no study comparing the “10% + blue” rule with other alternative criteria under SLNB using radioisotope and methylene blue in China.

Herein, we performed this retrospective analysis which included a large number of breast cancer patients with a prospectively constructed SLNB database at a single institution in China. We re-evaluated the “10% + blue” rule for breast cancer patients and sought to determine whether the threshold of hot nodes could be raised and what the impact it would be on both the accuracy and the number of lymph nodes excised when a higher than 10% threshold was used to define a SLN, potentially leading to patients with positive nodes being missed.

## Materials and Methods

This study was approved by our institutional review board.

### Study Population

Retrospectively, we reviewed the records of breast cancer patients who underwent SLNB successfully with a combination of radioactive colloid and methylene blue at our hospital from August 2010 to August 2019. Patients who were pathologically diagnosed with invasive breast cancer were eligible. Patients who received mastectomy for ductal carcinoma *in situ* (DCIS) were excluded. Patients who received neoadjuvant chemotherapy were also excluded. All patients were clinically node negative (negative in ultrasound, mammography, and physical examination) and had no regional or distant metastases.

### Surgical Techniques for SLNB

After the informed consent was obtained from each patient, Radioisotope -99mTc (Beijing Shihong Drug Development Center; Beijing, China) was injected intradermally at tumor surface and/or at periareolar site 3 to 18 h prior to the surgery, and methylene blue (Jiangsu Jichuan Pharmaceutical Co., Ltd; Jiangsu, China) was injected intradermally/subcutaneously at tumor surface and/or at periareolar site 10 to 15 min before surgery. During surgery, a hand-held gamma probe of 99mTc (Devicor Medical Products Inc.; OH, USA) was applied to identify SLNs. Any nodes with 10% or more of the *ex vivo* count of the hottest node and/or any nodes with at least one blue afferent lymphatic vessels derived from the breast were removed and designated as SLNs. Suspicious lymph nodes which were firm, enlarged and palpable but not radioactive or blue stained were also removed as non-SLNs. All nodes were evaluated with intraoperative frozen sections. ALND were performed based on the result of pathological evaluation. Generally, patients with SLNs of macrometastatasis (>2 mm) received ALND. It was recommended in the guidelines of China Anti-Cancer Association in 2017 that axillary dissection can be avoided in cT1-2N0 breast cancer patients who have 1 or 2 macrometastatic SLNs and are undergoing breast-conserving therapy and whole-breast radiation (7). Starting in 2018, for patients who meet the criteria of the ACOSOG Z0011, decisions to perform ALND or not should be made with full informed consent in our institution. Patients free of metastasis and those with SLNs of isolated tumor cells avoided further ALND. For patients with SLNs of micrometastatasis (>0.2 mm, ≤ 2 mm), decisions of ALND were made jointly by patients and the surgery group. Most of nodes removed were examined by permanent sections with hematoxylin-eosin (H&E) staining and immunohistochemical (IHC) staining for breast cancer-specific antigens if no macrometastasis was identified on routine assessment.

During surgery, the radioactivity, status of blue dye staining of nodes and lymphatic vessels, and the pathological results of each SLN were prospectively recorded so that we could calculate the number of SLNs identified by different criteria of radioactivity in combination with the status of blue staining.

### Statistical Analysis

In this study, we defined the rate of miss detection as the number of patients with positive nodes missed to be identified using alternative rules compared with the “10% + blue” rule divided by the total number of node-positive patients detected by the “10% + blue” rule. The chi-square test was used for categorical variables by SPSS 24 (SPSS Inc., Chicago, IL, USA). Figures were prepared by GraphPad Prism 8.0.1. Differences were considered significant at p ≤ 0.05.

## Results

A total of 1,039 invasive breast cancer patients successfully performed SLNB by dual tracers with the “10% + blue” rule. The clinical and pathologic characteristics of the study population were represented in **Table 1**.

**Table 1:** Clinical and pathologic characteristics of study population (n=1,039).

Variable	No.	%
Age, mean ± SD, y	48 ± 10.4	
≤40 y	243	23.4%
>40 y	796	76.6%
BMI, mean ± SD, kg/cm^2^	22.6 ± 2.9	
<24	759	73.1%
≥24	280	26.9%
Tumor location		
Upper inner quadrant	187	18.0%
Lower inner quadrant	70	6.7%
Upper outer quadrant	393	37.8%
Lower outer quadrant	130	12.5%
3 o’clock	14	1.3%
6 o’clock	17	1.6%
9 o’clock	68	6.6%
12 o’clock	60	5.8%
Central	66	6.4%
Unknown	34	3.3%
T stage (the AJCC, 8^th^ Edition)		
T1	625	60.2%
T2	391	37.6%
T3	23	2.2%
Histological type		
IDC	932	89.7%
Others^1^	107	10.3%
Hormone receptor status		
ER and/or PR positive	787	75.7%
ER and PR negative	215	20.7%
Unknown	37	3.6%
HER2 Status^2^		
Negative	533	51.4%
Positive	155	14.9%
Uncertain	311	29.9%
Unknown	40	3.8%
Ki-67 Status		
<15%	384	37.0%
15%-30%	297	28.6%
>30%	339	32.6%
Unknown	19	1.8%
Type of breast surgery		
Mastectomy	866	83.3%
Lumpectomy	173	16.7%
Type of axillary surgery		
SLNB only	810	78.0%
SLNB followed by ALND	229	22.0%

SD, standard deviation; AJCC, American Joint Committee on Cancer; IDC, invasive ductal cancer; DCIS, ductal carcinoma in situ; SLNB, sentinel lymph node biopsy; ALND, axillary lymph node dissection.

^1^including invasive lobular carcinoma, papillary carcinoma, mucous carcinoma, malignant phyllode tumor, secretory carcinoma, metaplastic carcinoma, squamous cell carcinoma, adenoid cystic carcinoma and mixed carcinoma.

^2^HER2 testing was performed by IHC and FISH if necessary. HER2 is positive when IHC is 3+ or IHC is 2+ with FISH is positive. HER2 is negative when IHC is 0-1+ or IHC is 2+ with FISH negative. HER2 is uncertain if IHC is 2+ without FISH. Her2 is unknown if IHC and FISH are unknown.

### Results of SLNB With “10% + Blue” Rule

**Figure 1:**
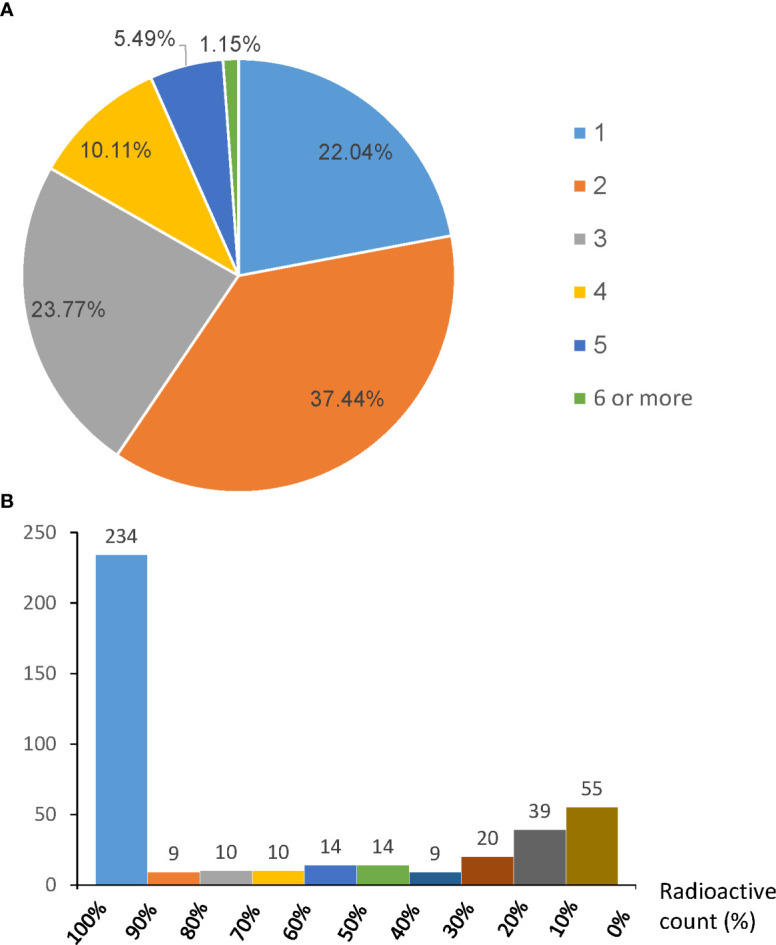
**(A)** Percentage of patients with different No. of sentinel lymph node (SLN) per patients identified by the “10% + blue” rule. **(B)** The radioactive count distribution of 414 positive SLNs by percentile of the hottest node. * Positive nodes with radioactive count percent <10% but blue staining.

A total of 2,529 SLNs were identified in 1,039 patients and 16.4% (414/2,529) SLNs were positive (micrometastases or macrometastases) (**Table 2**). A mean of 2.4 SLNs were identified. 78.0% (810/1,039) patients had at least two SLNs identified and 6.64% patients had five or more SLNs removed (**Figure 1A**). 121 non-SLNs were removed for enlarged and palpable but not blue or hot, of which 38 non-SLNs were positive. In a total of 309 patients with at least one positive axillary node (micrometastases or macrometastases), 296 patients had at least one positive SLN with or without positive non-SLNs and each of the remaining 13 patients had only one positive non-SLN. We do not know how many positive lymph nodes were missed due to the lack of complementary ALND, so the probability of non-SLN metastases in patients with SLN metastases (8.4%, 25/296) in this study was lower than that in the AMAROS trial and the Z0011 trial which had approximately one-third patients with a positive non-SLN in the ALND group (12, 13). Among the 414 positive SLNs, 70.3% (291/414) had a radioactivity count of 40% or more than the hottest node and 13.3% (55/414) were blue stained with a less than 10% radiation count of the hottest node (**Figure 1B**). Among 2,115 negative SLNs, 1,413 nodes were blue stained while up to 1,792 were radioactively hot, leading to 17.9% (379/2,115) negative nodes being excessively excised as radioactively hot nodes. Numbers of positive and negative SLNs detected by radioactive colloid and blue dye were shown in **Table S1–3**, respectively.

**Table 2:** Outcomes of the dual tracer using a combination of blue dye and radioactive colloid with the 10% criteria.

Characteristics	No.	%
SLN identified by dual tracers	2,529	
mean number of SLNs identified, mean ± SD	2.4 ± 1.16	
positive SLN	414	16.4%
detected by blue dye	333	
detected by the radioactive colloid tracer with 10% rule	359	
negative SLN	2,115	83.6%
detected by blue dye	1,413	
detected by the radioactive colloid tracer with 10% rule	1,792	
non-SLN	121	
Positive non-SLN	38	
Negative non-SLN	83	
Patients with negative axillary nodes	730	70.3%
Patients with positive axillary nodes	309	29.7%
≥1 positive SLN with or without positive non-SLNs	296	
Only one positive non- SLN	13	
Patients with only one SLN identified	229	22.0%
Patients with two or more SLNs identified	810	78.0%

### Different Alternative Rules Compared With “10% + Blue” Rule

**Figure 2:**
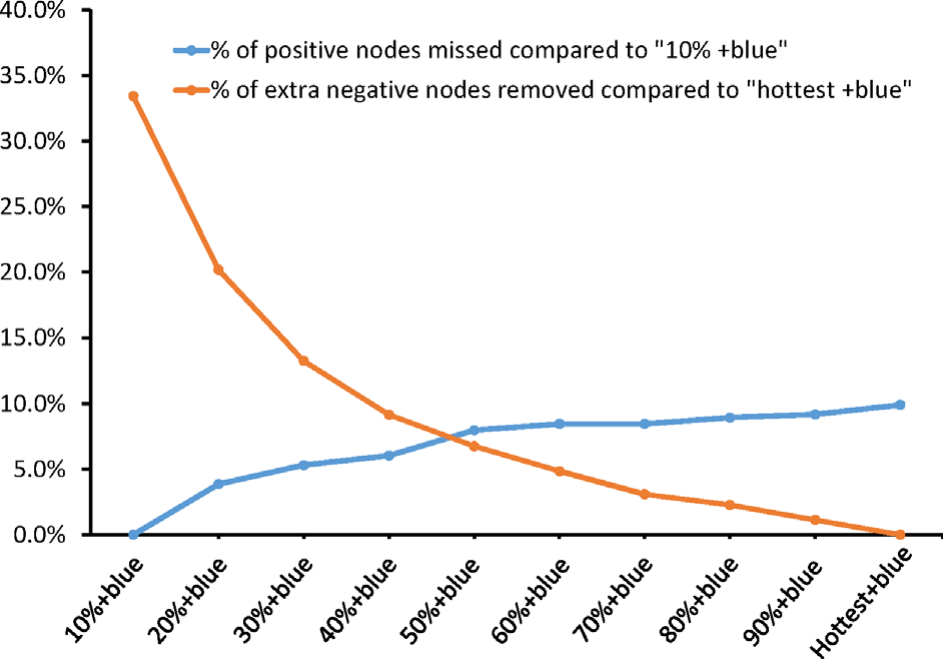
Positive nodes missed and extra negative removed by combing different percentage rules with blue dye.

Different percentile rules for radioactivity were compared with the “10% + blue” rule (**Table 3**). As is shown in **Figure 2**, the balance between fewer positive nodes missed and more negative nodes reserved was between the “40% + blue” rule and the “50% + blue” rule. From the “10% rule + blue” rule to the “50% + blue” rule, the average number of SLNs identified per patients dropped from 2.43 to 2.00. Compared with the “10% + blue” rule, when the “40% + blue” rule was applied, the rate of positive SLNs increased from 14.80% to 16.58% (p>0.05) and negative SLNs decreased by 18.2% (385/2,115), resulting in a rate of miss detection of only 1.00% (3/296). If only the hottest or blue nodes were removed, seven patients with positive nodes would be undetected, resulting in a rate of miss detection was 2.7%. Characteristics of the seven patients were shown in **Table S4**. There was no statistically significant difference found with respect to the rate of positive SLNs and the rate of miss detection when applying the criteria anywhere from 10% to the hottest for identifying SLNs compared with the “10% + blue” rule.

**Table 3:** Effect of different percentage criteria on sentinel lymph node (SLN) identification positive SLN (n=1,039).

Rules	Blue SLNs	Hot SLNs	Hot and blue SLNs	SLNs detected by dual tracers	Detection rate of overall SLNs (per-SLN)	% of nodes reduced	Positive SLNs	Detection rate of positive SLNs (per-SLN)	Patients with positive SLNs	Miss rate of positive SLNs (per-patient)^1^	Detection rate of overall SLNs (per-patient)
≥10%+blue	1,746	2,151	1,368	2,529	ref	ref	414	ref	296	ref	100%
≥20%+blue	1,746	1,779	1,222	2,303	91.1%	8.9%	398	96.1%	293	1.0%	100%
≥30%+blue	1,746	1,568	1,127	2,187	86.5%	13.5%	392	94.7%	293	1.0%	100%
≥40%+blue	1,746	1,435	1,062	2,119	83.8%	16.2%	389	94.0%	293	1.0%	100%
≥50%+blue	1,746	1,342	1,015	2,073	82.0%	18.0%	381	92.0%	291	1.7%	100%
≥60%+blue	1,746	1,257	962	2,041	80.7%	19.3%	379	91.5%	291	1.7%	100%
≥70%+blue	1,746	1,190	923	2,013	79.6%	20.4%	379	91.5%	291	1.7%	100%
≥80%+blue	1,746	1,138	886	1,998	79.0%	21.0%	377	91.1%	290	2.0%	100%
≥90%+blue	1,746	1,087	854	1,979	78.3%	21.7%	376	90.8%	289	2.4%	100%
hottest+blue	1,746	1,034	822	1,958	77.4%	22.6%	373	90.1%	289	2.7%	100%
blue only	1,746	–	–	–	69.0%	31.0%	333	80.4%	261	11.8%	90.4%
≥10% only	–	2,151	–	–	85.1%	14.9%	359	86.7%	273	7.7%	99.4%

^1^Miss rate of positive SLNs on a per-patient basis= 1- Detection rate of positive SLNs on a per-patient basis = (296-patients with positive nodes detected by each threshold)/296.

Finally, we assessed the “hottest two + blue” rule in 810 patients with at least two SLNs identified by the “10% + blue” rule in this study. The outcomes were presented in **Table 4**. Compared to the “10% + blue” rule, 23 positive nodes were undetected causing 0.84% (2/239) patients with positive nodes missed whereas 12.26% (238/1,942) negative nodes were reserved. Of note, among the 23 positive nodes missed to be identified, 3 nodes were from two node-positive patients who would have been missed to be detected using the “hottest two + blue” rule, and other 20 nodes were from 20 node-positive patients who could have been identified using the “hottest two + blue” rule.

**Table 4:** Effect of different criteria on sentinel lymph node (SLN) identification in patients with two or more SLNs removed (n=810).

Rules	SLNs with blue staining	Hot SLNs	SLNs detected by dual tracers	Positive SLNs	Negative SLNs	Patients with positive SLNs	Patients with negative SLNs	% of negative nodes reserved	No. of SLNs per patients	Miss rate
Blue dye	1,553	–	–	286	1,267	214	596	–	1.92	10.5%
10% rule	–	1,926	–	303	1,623	216	594	–	2.38	9.6%
10%rule+blue	1,553	1,926	2300	358	1,942	239	571	Ref	2.84	ref
Hottest	–	810	–	169	639	169	641	–	1.00	29.3%
Hottest two	–	1,620	–	271	1,349	218	592	–	2.00	8.8%
Hottest two + blue	1,553	1,620	2039	335	1,704	237	573	12.3%	2.52	0.8%

## Discussion

During the last decades, the concept of the treatment strategy for breast cancer has shifted from maximum tolerated therapy to minimum effective therapy. With the improvement of imaging examination and the popularization of screening, breast cancers diagnosed at early stage have strongly increased (14–17). In non-surgical area, improvements in multimodal therapy, including advances in modern radiotherapy technology, optimization of chemotherapy, and anti-HER2 therapy regimens, novel endocrine agents, and target drugs, as well as clinical utility of immunotherapy, could further diminish breast cancer mortality and contribute to increase chances for cure in 70%–80% patients with early breast cancer (18, 19). In large clinical trials such as the AMAROS and the ACOSOG Z0011, the residual tumor burden from limited metsastatic nodes may be further reduced, resulting in an extremely low recurrence rate (<2%) (12, 13, 20). With extended survival, the quality of life is becoming more important. The dual tracer combining radioisotope and blue dye remains the mainstream in the current clinical routine, especially in institutions where materials and equipment for new tracing method are not available. Exploring optimized criteria based on the dual-tracer method is more conducive to improve the quality of life for a wide range of patients. Therefore, in this study, we merely focused on the dual tracer method of radioactive colloid and methylene blue, rather than other new techniques for SLNB such as indocyaninegreen.

Although SLNB is associated with improved quality of life and reduced arm morbidities without compromising the survival in patients with early stage breast cancer compared to ALND (21, 22), a considerable number of patients undergoing SLNB still suffer from arm and shoulder impairments. Prevalence of lymphedema one year after SLNB ranges between 3% and 17% and for pain, prevalence between 3.3% and 56.6% have been reported in SLN-negative breast cancer patients (23–25). Some studies reported that a greater number of nodes removed, especially more than ten nodes dissected, was associated with an increased risk of lymphedema in ALND patients (26–28) although existing studies failed to find this association in SLNB patients (24, 29, 30). However, the observation that the arm morbidity occurs in a certain proportion of patients who received SLNB leads us to worry that a larger number of SLNs dissected may contribute to a higher risk of arm morbidity. In this study, 16.4% nodes were harvested for metastases at an expense of 83.6% negative nodes removed excessively. Furthermore, up to 17.9% negative nodes were removed as radioactively hot nodes without blue staining. Besides, 6.64% patients had five or more SLNs removed, which may weaken the advantage of SLNB as a less invasive procedure. The more SLNs removed, the higher the cost of the procedure for added time during surgery and increased pathological charges. When no metastases are detected by routine H&E, more in-depth histologic evaluation such as IHC will be applied to detect (micro-)metastases, making the procedure more expensive than routine histology (31, 32).

Is there a more reasonable guide for identifying SLNs with less unnecessary nodes removed for breast cancer? To our knowledge, several previous retrospective studies compared the dual tracer using 10% rule with various blue dye and a few studies attempted to seek alternative methods. Nagao et al. assessed the “10% + blue” rule and the “4 node” rule by involving 302 patients with Tis-T3 breast cancer who underwent SLNB with a combination of radioisotope and indigo carmine blue dye and concluded that terminating SLNB at the first three SLNs identified all node positive patients with a low false negative rate (FNR) and rate of complication (9). In a study of 475 patients with T1-2 breast cancer who underwent SLNB with a combination of radioisotope (10% rule) and blue dye (lymphazurin or methylene), Dutta et al. indicated that no more than 4 SLNs should be removed because all patients with positive nodes were identified within the first 4 SLNs removed (10). Liu et al. studied 332 patients with T1-T3 breast cancer who underwent SLNB and showed that using the “40%” rule as the criteria for removal of SLN resulted in a 10.3% FNR and “10%” rule resulted in a 6.4% FNR; however, surgeons selectively used lymphazurin blue so the radioisotope was generally used alone in the study (11). Another large retrospective study involving 6519 patients with T1-T3 breast cancer who underwent SLNB with a combination of radioisotope and isosulfan blue dye performed by Chung et al. reported that the “10% +blue” rule was a reliable guideline but they didn’t determine other potential percentile cut-off of hot nodes (33). We first built the model by gradually increasing the percentile threshold of radioactivity count in a large prospectively collected database of breast cancer patients who performed SLNB by dual tracers of methylene blue and radioisotope in China. Our data demonstrated that compared with the “10% + blue” rule, the number of nodes identified would reduce by 16.2% at a cost of only three positive patients being missed (1.0%) when the “40% +blue” rule was used. Similarly, in patients with at least two SLNs removed, 12.3% negative nodes were able to avoid being removed unnecessarily with only 0.8% positive patients missed by the “hottest two + blue” criteria. The potential 16.2% and 12.3% reduction in nodes that need pathological examination may offer a considerable cost-effectiveness benefit of the procedure. Our result revealed that replacing the “10% +blue” rule with the “40% +blue” rule or the “hottest two + blue” rule can be considered as a potential alternative model to minimize extra negative nodes removed without significantly increasing the number of node positive patients missed.

The main concern for patients with SLNB is the impact of missed nodes on locoregional recurrence and survival. In the NSABP B-06 trial which was designed to determine whether SLNB achieve an equivalent survival and regional control as ALND, breast cancer patients with negative SLNs were randomly assigned 1:1 to ALND or SLNB alone. It reported that each group had less than 1% regional node recurrences as first events by eight years (ALND group vs SLNB group: 8/1,975 vs 12/2,011, p=0.22) with 9.8% FNR in the ALND group (34). The Milan trial also showed that 2 patients in the SLNB group developed axillary recurrence with 8 patients estimated to have occult axillary involvement (35). Besides, in the AMAROS trial and the Z0011 trial, the axillary recurrence was extremely low (<1%) in the SLNB group with an estimated one-third residual lesions (12, 13). In our study, only 0.29% (3/1,039) node-positive patients were missed when we changed the “10% +blue” rule to the “40% +blue” rule and 0.25% (2/810) when we replaced the “10% +blue” rule with the “hottest two + blue” rule. In the era of subsequent effective and complete adjuvant therapy, the residual lesions may be further reduced. We therefore would expect to see an extremely low recurrence rate.

Some limitations of this study should be mentioned. First, in this retrospective study, ALND was not performed in patients with negative SLNs because of ethical issues. A small number of SLN-positive patients chose to avoid further ALND starting in 2018, which was a bit behind the clinical trials and guidelines. Therefore, the actual sensitivity, specificity, accuracy and FNR of SLNB were unlikely to be calculated. What we were most concerned about was the FNR of alternatives to the “10% + blue” rule. Thus we defined the term “the rate of miss detection” similar to Liu and Murphy (36, 37) and no statistically significance was found anywhere from the “10% +blue” rule to the “hottest + blue” rule. Besides, in our institution, to ensure a low FNR within 5%, at least 40 cases were required for the learning curve for SLNB before surgeons could contribute to this database so that our conclusion could not be affected by the shortcoming of unknowing true FNR. Second, patients with micrometastatic SLNs were offered observation or ALND with full informed consent, which was somewhat behind the latest guidelines and the IBCSG 23-01 trial which indicated that ALND should be avoided in SLN-micrometastatic patients receiving breast-conserving surgery (38). Besides, we didn’t group patients prospectively and the study was a single monocentric experience without confirmation in an external dataset, so we did not know the local control of patients undergoing SLNB with different criteria. Though the effect of missing positive patients on survival was not expected to be great according previous literature as discussed above, the results of this study should be validated by multiple-center prospective studies with long-term follow up for prognosis.

## Conclusions

Our data demonstrated that the “40% + blue” rule or the “hottest two + blue” rule can be considered as a potential alternative model to minimize extra negative nodes removed without significantly increasing the number of node-positive patients missed. The results should be further validated in prospective clinical trials with long-term follow up.

## Data Availability Statement

The raw data supporting the conclusions of this article will be made available by the authors, without undue reservation.

## Ethics Statement

The studies involving human participants were reviewed and approved by Westchina hospital, Sichuan University. The patients/participants provided their written informed consent to participate in this study.

## Author Contributions

Study conception and design: QL. Data collection: ZD, FL, YX, QYL, JC, and HZ. Data analysis: LX and JY. Data interpretation: QL, ZD, LX, and JY. Manuscript preparation: LX and JY. All authors contributed to manuscript revision, read, and approved the submitted version.

## Funding

This study is supported by the funding from the National Natural Science Foundation of China (81902686); China Postdoctoral Science Foundation Funded Project (2019M663511); the Post-Doctor Research Project, West China Hospital, Sichuan University (2019HXBH046); the Program of the Science and Technology Bureau of Sichuan (2020YJ0293, 2019YFH0146 and 2020YFS0199); and Chengdu Science and Technology Bureau (2019-YF05-01082-SN).

## Conflict of Interest

The authors declare that the research was conducted in the absence of any commercial or financial relationships that could be construed as a potential conflict of interest.
