# Supplementary material for: Redefining Criteria to Ensure Adequate Sentinel Lymph Node Biopsy With Dual Tracer for Breast Cancer
Source: Front Oncol. 2020 Dec 3;10:588067. doi: 10.3389/fonc.2020.588067 (PMC7744718; doi:10.3389/fonc.2020.588067)
Supplement: Supplementary file 1 [file DataSheet_1.docx]

***Supplementary Material***

**1 Supplementary Figures and Tables**

**1.1 Supplementary Tables**

**Supplementary Table 1** Negative SLNs detected by dual tracers

**Supplementary Table 2** Positive SLNs detected by dual tracers

**Supplementary Table 3** Patients with positive SLNs detected by dual tracers

**Supplementary Table 4** Seven patients with positive nodes missed if only the hottest node being removed

**1.1 Supplementary Tables**

**Supplementary Table 1** Negative SLNs detected by dual tracers

| Blue dye | Radioactive colloid tracer (10%) | | Total SLNs |
| --- | --- | --- | --- |
|  | hot | not hot |  |
| Blue | 1090 | 323 | 1413 |
| Not blue | 702 | 0 | 702 |
| Total SLNs | 1792 | 323 | 2115 |

**Supplementary Table 2** Positive SLNs detected by dual tracers

| Blue dye | Radioactive colloid tracer (10%) | | Total SLNs |
| --- | --- | --- | --- |
|  | hot | not hot |  |
| Blue | 278 | 55 | 333 |
| Not blue | 81 | 0 | 81 |
| Total SLNs | 359 | 55 | 414 |

**Supplementary Table 3** Patients with positive SLNs detected by dual tracers

| Blue dye | Radioactive colloid tracer (10%) | | Total Patients |
| --- | --- | --- | --- |
|  | hot | not hot |  |
| Blue | 238 | 23 | 261 |
| Not blue | 35 | 0 | 35 |
| Total Patients | 273 | 23 | 296 |

| **Table S4** Seven patients with positive nodes missed if only the hottest node being removed | | | | | | |
| --- | --- | --- | --- | --- | --- | --- |
| No. of patient | ex vivo radioactive count of positive node | ex vivo radioactive count of the hottest node | Percent of hottest node | Order of radioactive count | Blue staining | Status of SLN ^1^ |
| 1 | 160 | 1131 | 14.10% | 3rd of 3 | None | Macrometastasis by frozen sections |
| 2 | 377 | 799 | 47.18% | 2nd of 3 | None | Micrometastasis by permanent sections |
| 3 | 237 | 329 | 72.04% | 2rd of 3 | None | Macrometastasis by frozen sections and permanent sections |
| 4 | 125 | 133 | 93.98% | 2nd of 2 | None | Macrometastasis by frozen sections and permanent sections |
| 5 | 202 | 244 | 82.79% | 2nd of 3 | None | Micrometastasis by permanent sections |
| 6 | 116 | 875 | 13.26% | 2nd of 4 | None | Macrometastasis by permanent sections |
| 7 | 115 | 842 | 13.66% | 3nd of 3 | None | Macrometastasis by permanent sections |
| ^1^ Metastatic lesions over 2.0 mm were classified as macrometastasis:; metastatic lesions between 0.2 mm and 2 mm were classified as micrometastasis:; isolated tumor cells (metastatic lesions less than 0.2 mm) were considered to be not metastasis. | | | | | | |
